# VDR mRNA Expression And Serum Vitamin D Levels in Post-Covid Vaccinated Patients

**DOI:** 10.12688/f1000research.144077.2

**Published:** 2026-02-14

**Authors:** Sandesh Shende, Jaishriram Rathored

**Affiliations:** 1 Clinical Research, Datta Meghe Institute of Higher Education and Research, Sawangi Meghe, Maharashtra, 442001, India; 2 Central Research laboratory (CRL) and Molecular Diagnostics, Datta Meghe Institute of Higher Education and Research, Sawangi Meghe Wardha, Maharashtra, 442001, India

**Keywords:** Vitamin D Receptor, mRNA expression, COVID-19, intact Parathyroid Hormone, Calcium level, Serum vitamin D

## Abstract

The emergence of COVID-19 vaccines has reshaped the trajectory of the ongoing pandemic, offering hope for widespread immunity. Beyond conferring protection against SARS-CoV-2, these vaccines have exhibited intriguing immunomodulatory effects. This research explores the dynamic interplay among VDR mRNA expression levels, calcium (ionized and total), and intact parathyroid hormone (iPTH) concentrations in individuals post-COVID-19 vaccination.

The Vitamin D Receptor (VDR) plays a pivotal role in immune regulation and is closely intertwined with calcium homeostasis. This study investigates the hypothesis that COVID-19 vaccination may induce alterations in VDR mRNA expression, subsequently influencing calcium metabolism and iPTH secretion.

Our findings reveal dynamic shifts in VDR mRNA expression following COVID-19 vaccination, with distinct patterns observed across individuals. Concurrently, we observe ionized and total calcium levels alterations, hinting at potential links between VDR activity and calcium metabolism post-vaccination. Furthermore, iPTH levels exhibit intriguing fluctuations, suggesting a regulatory role of VDR in parathyroid hormone secretion.

The integration of clinical outcomes and vaccine response data sheds light on the significance of these molecular and biochemical alterations.

This research underscores the multifaceted impact of COVID-19 vaccination on VDR mRNA expression, calcium homeostasis, and iPTH regulation. Beyond the scope of vaccination, our findings may bear implications for immunomodulation in various disease contexts, particularly in individuals with pre-existing calcium-related disorders.

In conclusion, our study unveils the intricate relationships among VDR mRNA expression, calcium levels, and iPTH concentrations in the context of post-COVID-19 vaccination. These discoveries extend our understanding of vaccine-induced immunomodulation and may pave the way for personalized vaccination strategies, while also opening new avenues for investigating the role of VDR in immune responses and calcium regulation beyond the pandemic.

## Introduction

In the global pursuit of ending the COVID-19 pandemic, vaccination campaigns have emerged as a beacon of hope, ushering in a new era of resilience and recovery. While vaccines have played a main role in mitigating the devastating impact of virus, ^1^ they have also prompted numerous questions about their effects on the human body, especially in molecular biology. ^2^ Among the myriad questions that have arisen, one has particularly intrigued scientists and clinicians alike: How do COVID-19 vaccines impact the expression of the Vitamin D Receptor (VDR) mRNA in the human body, and what could this mean for our understanding of immune responses and overall health?

The COVID-19 outbreak has challenged healthcare professionals throughout the world with challenges that have never been encountered before. ^3^ Numerous lives have been saved as a result of the vital role that quick vaccine development and distribution have played in controlling the virus’s spread. ^4^ But there is still much to learn about the complex interactions between vaccinations, the immune system’s function, and other biological functions. ^5^

The VDR, a vital part of the immune system in humans, is well-known for its crucial function in controlling immunological responses, regulating pathways of inflammation, and maintaining general health. ^6^ Vitamin D, also known as “sunshine vitamin,” plays a crucial part in regulating VDR expression. ^7^^–^^9^ Variations in VDR mRNA levels of expression may affect how each person reacts to vaccines and viral illnesses, according to recent research. ^10^

Gaining a thorough grasp of how COVID-19 immunization affects VDR mRNA expression is crucial as the globe struggles with COVID-19 and its continuous mutations. ^11^ This information could provide insight into the long-term implications of side effects as well as the processes underlying them. Additionally, it can provide insightful information on individualized vaccination plans and the creation of innovative treatments to improve vaccine responses.

In this investigation, we set out to understand the complex molecular dynamics of VDR mRNA expression in post-COVID-vaccinated patients. I hope to give a comprehensive view of how COVID-19 vaccinations affect the expression of this important receptor by diving into the worlds of molecular biology, and immunology. In order to understand the molecular symphony that directs our immune response to COVID-19 immunization, our work makes use of cutting-edge methodologies, extensive data analysis, and multidisciplinary collaboration.

I want to further my knowledge of the intricate relationships among vaccines, the immune system, and human genetics through this endeavour, bridging the gap between scientific curiosity and useful healthcare applications. As we journey through the labyrinthine pathways of VDR mRNA expression, my goal is not only to shed light on its modulation but also to illuminate new avenues for harnessing this knowledge to strengthen our defences against current and future pandemics.

## Review of literature

### The role of Vitamin D and VDR in immunity

Understanding the interplay between Vitamin D and the VDR is crucial in deciphering the complexities of post-vaccination immune responses. ^12^ Vitamin D is known for its pleiotropic effects on the immune system, and the VDR serves as the key mediator in this process. ^13^ The modulation of innate and adaptive immunological responses, cytokine synthesis, and T-cell activity have all been connected to vitamin D. ^6^ Therefore, any changes in VDR expression may have profound effects on immunological homeostasis. ^6^ The synthesis of calcitriol in monocytes and macrophages is likely necessary for vitamin D to exert immunomodulatory effects. ^14^ To completely comprehend the overall impact of vitamin D on the human immune system, further questions need to be addressed. ^6^

### Variability in VDR mRNA expression

Several studies have demonstrated the variable expression of VDR mRNA in different tissues and among individuals. Environmental factors, such as polymorphisms in VDR gene and exposure to sunlight, contribute to this variability. ^15^ However, the specific impact of COVID-19 vaccination on VDR mRNA expression patterns remains a relatively unexplored territory. ^16^ Investigating how vaccination influences these expression patterns is essential for understanding vaccine responsiveness and potential long-term effects. ^17^

### Immunological consequences of altered VDR expression

Altered VDR mRNA expression may affect immune responses in multiple ways. ^18^ It may influence the production of antimicrobial peptides, cytokines, and chemokines. ^19^^,^^20^ Understanding these consequences could provide insights into vaccine efficacy and the risk of immune-related adverse events, offering guidance for tailored vaccination strategies. ^21^

### Therapeutic potential

Exploring the modulation of VDR mRNA expression could open new avenues for therapeutic interventions. ^22^ Fine-tuning VDR expression may offer ways to enhance vaccine responses in specific populations or mitigate adverse effects. ^23^ Moreover, VDR agonists have been investigated for their potential in managing inflammatory and autoimmune conditions, making this receptor an intriguing target for future research. ^24^

## Research gap analysis

### Limited comprehensive studies

One of the primary research gaps in the field is the paucity of comprehensive studies examining VDR mRNA expression following COVID-19 vaccination. ^25^ There is a pressing need for large-scale, multi-center studies encompassing diverse populations to capture the full spectrum of VDR expression changes post-vaccination. ^26^

### Temporal dynamics

While some studies have provided insights into VDR expression shortly after vaccination, there is a notable lack of research investigating the temporal dynamics of VDR mRNA expression. ^27^ Understanding how VDR expression evolves over time, especially in the context of booster shots and potential long-term effects, is crucial for informed vaccination strategies. ^28^

### Tissue-specific variation

The VDR is expressed in a tissue-specific manner, with varying levels in different organs and cell types. ^29^ Yet, research often overlooks the importance of tissue-specific analysis. A comprehensive examination of VDR mRNA expression across relevant tissues, such as immune cells, respiratory epithelium, and lymphoid organs, is essential to grasp the full immunological implications of vaccination. ^30^

### Functional correlates

While changes in VDR mRNA expression are intriguing, their functional consequences remain underexplored. ^31^ Investigating the downstream effects of altered VDR expression on immune responses, and cytokine profiles is paramount to connect molecular changes with clinical outcomes. ^32^

### Environmental factors

Environmental factors like sunlight exposure are known to influence VDR expression. ^33^ However, research often neglects these variables. Examining how these factors interact with vaccination-induced changes in VDR mRNA expression could provide insights into personalized vaccine responses.

### Comparative studies

Comparative studies between different COVID-19 vaccines and vaccine platforms are scarce. ^34^ Investigating whether different vaccines induce distinct patterns of VDR mRNA expression could inform vaccine selection and development strategies. ^35^

### Therapeutic implications

While some studies hint at the therapeutic potential of modulating VDR expression, ^36^ there is a dearth of research specifically designed to harness this knowledge for clinical applications. ^37^ Developing targeted interventions based on VDR expression changes could revolutionize vaccine strategies and benefit populations with suboptimal responses. ^23^

**Research questions:** Whether there is difference in serum vitamin D, serum calcium and iPTH level in post vaccinated individuals with booster dose and as compared to without booster dose (Covishield)? Whether there is correlation between vitamin D level, iPTH, Calcium, mRNA with respect to linear regression analysis?

## Hypothesis

### Research hypothesis

To study the VDR mRNA expression in post covid vaccinated patient and correlate with different biochemical parameters like Vit D level, Intact Parathyroid Hormone (iPTH) and calcium level (ionized and total) and try to rule out whether this mRNA expression is responsible for deficiency of Vit D and susceptible for covid infection.

### Aim of the study

To evaluate the impact of COVID-19 vaccination status (booster-vaccinated vs partially vaccinated) on Vitamin D receptor (VDR) mRNA expression, serum Vitamin D, calcium, and intact parathyroid hormone (iPTH) levels, and to determine corelation in post-vaccinated individuals.

### Objectives

1. (a) To quantify VDR mRNA expression, serum Vitamin D, serum calcium, and iPTH levels in fully vaccinated with booster dose of Covishield.
(b) To quantify VDR mRNA expression, serum Vitamin D, serum calcium, and iPTH levels in partially vaccinated without booster dose of Covishield.
2. To study the corelation between VDR mRNA expression, serum Vitamin D, calcium and iPTH levels in both the groups (with booster and without booster dose of Covishield).
3. To compare the VDR mRNA expression, serum Vitamin D levels, calcium homeostasis and iPTH regulation in both the groups (booster vs no booster).

**Figure:**
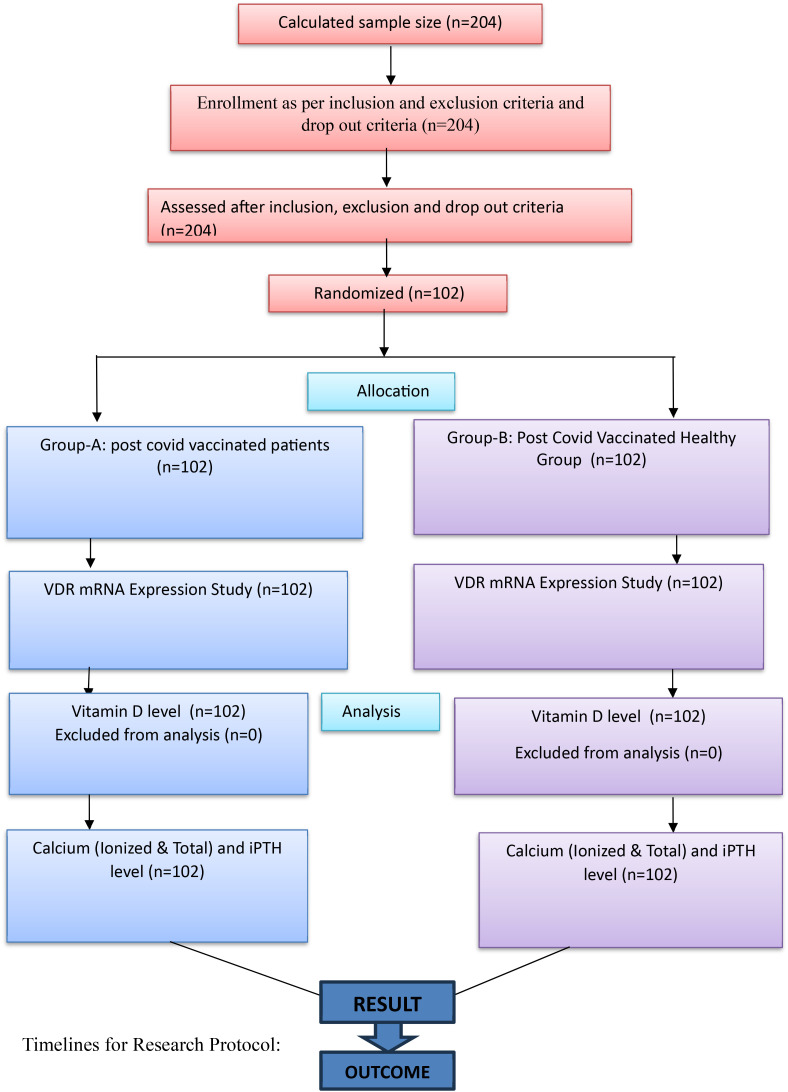
Schematic diagram of study design.

## Methods

**Study design:** Cross-sectional study

**Place of study:** Acharya Vinoba Bhave Rural Hospital (AVBRH), Sawangi (Meghe), Wardha

**Informed consent form (ICF):** All participants will be enrolled after obtaining ICF.

**Participant recruitment:** Participants will be recruited based on strict exclusion and inclusion criteria mentioned below

### Inclusion criteria

1. Adults aged 18–65 years.
2. Individuals who have received COVID-19 vaccination exclusively with Covishield.
3. Participants were grouped into:
  I. Booster-vaccinated group: Individuals who have completed two doses of Covishield vaccination and received a booster dose (3rd Dose).
  II. Partially vaccinated group: Individuals who have completed primary Covishield with one or two doses of vaccination without receiving any booster dose (3rd Dose).
4. Apparently healthy individuals without acute illness or any infection at the time of recruitment.
5. Willingness to participate and provide written informed consent.

### Exclusion criteria

1. History of confirmed COVID-19 infection within the past 6 months.
2. Prior vaccination with any COVID-19 vaccine other than Covishield.
3. Current or recent (<3 months) use of Vitamin D or calcium supplements.
4. Known disorders affecting calcium or Vitamin D metabolism: Chronic kidney disease, Chronic liver disease, Parathyroid disorders, Osteoporosis or metabolic bone diseases.
5. Use of medications influencing Vitamin D or calcium metabolism: Corticosteroids, Antiepileptic’s, Hormone replacement therapy.
6. Autoimmune diseases, malignancy, or chronic inflammatory conditions.
7. Pregnancy or lactation.
8. Individuals with recent major surgery or hospitalization within the past 3 months.
9. Any acute infection or febrile illness at the time of sample collection.

**Healthy subjects** will be recruited from the general population with the same socioeconomic status and those who are free from any diseases and comorbidities.

### Data collection

**Demographic and clinical data:** Structured questionaries will be used to collect demographic information, vaccination history, and relevant medical history from each participant.

**Blood sampling:** Peripheral blood samples (5-10 ml) will be obtained from participants by a trained phlebotomist.

### VDR mRNA expression analysis

**Isolation of peripheral blood mononuclear cells (PBMCs):** PBMCs will be collected from whole blood with the help of density gradient centrifugation protocol as per manufacturer instructions.

**RNA isolation:** It will be done by Insta NX Mag16 automated Nucleic acid extractor machine and RNA Extraction kits, HiMedia Lab, India, followed by manufacturer’s instructions.

**cDNA synthesis and Real-time PCR:** cDNA synthesis will be done in RT-PCR machine (QuantStudio ^TM^ 5 Real-Time PCR System, Thermo Fisher Scientific, Waltham, Massachusetts, USA) followed by reverse transcriptase PCR. After cDNA synthesis real-time PCR will be done as per manufacturer instructions followed by RTPCR kits.

### Environmental factors

**Sunlight exposure assessment:** Sunlight exposure history was assessed through participant questionnaires, including information on average daily sun exposure, and sunscreen use.

### Data management and statistical analysis

**Data storage:** All data, including demographic, clinical, and molecular data, were securely stored in Excel to ensure data integrity and participant confidentiality. Double data entry will be done to maintain the quality and integrity.

**Statistical analysis:** For Statistical analysis, all data will be presented as mean standard deviation (SD) CV % and P-values for statistical significance. Comparison between Post Vaccinated COVID-19 patients with Healthy subjects will be done using unpaired T-test. All analyses will be performed using statistical SPSS software.

**Participant confidentiality:** All participants’ personal information and data will be kept confidential.

**Informed consent:** Informed consent procedures will be followed, and participants will be informed about the study’s purpose, risks, and benefits.

**Ethics approval:** Applied and will be obtained before the study starts.

### Timeline

The study will be completed in 3 years, including participant recruitment, data collection, laboratory analyses, and data analysis.

**Table:** Timelines for research protocol.

S. no	Quarterly Scheduling of Research work	Date of Initiation	Duration of Activity
1.	Choosing the research question in tune with recent developments and new challenges in the Preparation of VDR mRNA expression and serum Vit. D levels in Post Covid vaccinated patients. Relevant literature search establishing hypothesis and preparation of synopsis.		6 months
2.	The availability of laboratory research support. Submission of synopsis for IEC clearance.		1 Year
3.	Assessment of subject data Sample collecting and Processing of Research in CRL		1 Year
4.	Data Analysis and writing of thesis		6 months

### Results presentation

The findings will be presented in the form of tables, graphs, and figures related to VDR mRNA expression and different biochemical other potential parameters used in the present study.

### Discussion and interpretation

The findings will be discussed in the context of existing literature, and implications and will be able to draw the relevant conclusion.

### Sample size calculation

Formula:

$$n\geq \frac{Z_{1-\alpha /2}^{2}\times p\left(1-p\right)}{d^{2}}$$

Where: *Z* = 1.96 for a confidence level (α) of 95%,

*p* = Prevalence of Covid 19 vaccination in India 95%

*e* = margin of error.

Z=1.96,p=0.95,e=0.03

$$n={\left(1.96\right)}^{2}\;*\;0.95\;*\;\left(1-0.95\right)/{\left(0.03\right)}^{2}$$

*n* = 203

The sample size is 203 total subjects

(for equal distribution of cases and control we will take 204 sample size) ^38^

IEC Approval: Got the approval of my Synopsis from Datta Meghe Institute of Higher Education and Research. IEC Approval Letter No. is DMIHER (DU)/IEC/2023/56.

Trial Registration: Applied for Registration on CTRI website and Reference No. is. REF/2024/01/077535 AU.

### Outcomes

*Primary outcome*

To identify the possible mechanism of VDR signalling molecules through mRNA expression leads to Vitamin D deficiency in post vaccinated covid patients.

*Secondary outcome*

To address the factors responsible for defective VDR signalling of post vaccinated covid patients through different biochemical parameters (Vitamin D, Total Calcium, Ionized calcium, iPTH hormone).

## Data Availability

No data are associated with this article.
