# Supplementary material for: VDR mRNA Expression And Serum Vitamin D Levels in Post-Covid Vaccinated Patients
Source: F1000Res. 2026 Feb 14;13:349. Originally published 2024 Apr 23. [Version 2] doi: 10.12688/f1000research.144077.2 (PMC12914166; doi:10.12688/f1000research.144077.2)
Supplement: Peer review history

# Reviewer report: Reviewer response for version 2

**DOI:** 10.5256/f1000research.196483.r458588

**Published:** 2026-02-17

**Authors:** Olga Adriana Caliman-Sturdza

**Affiliations:** 1 Stefan cel Mare University of Suceava, Suceava, Romania

The authors revised the manuscript following the comments raised and answered all my questions.

Is the study design appropriate for the research question?

Partly

Is the rationale for, and objectives of, the study clearly described?

Partly

Are sufficient details of the methods provided to allow replication by others?

Partly

Are the datasets clearly presented in a useable and accessible format?

Partly

Reviewer Expertise:

Infectious Diseases, Immunology, Viral Diseases

I confirm that I have read this submission and believe that I have an appropriate level of expertise to confirm that it is of an acceptable scientific standard.

## References

[1] : Vitamin D and COVID-19: Clinical Evidence and Immunological Insights. *Life* .2025;15(5) : 10.3390/life15050733 10.3390/life15050733 PMC1211280640430160

# Reviewer report: Reviewer response for version 1

**DOI:** 10.5256/f1000research.157818.r398540

**Published:** 2025-07-25

**Authors:** Olga Adriana Caliman-Sturdza

**Affiliations:** 1 Stefan cel Mare University of Suceava, Suceava, Romania

Thank you to the authors for sending me this study protocol for review. The topic could be interesting, but there are some ambiguities regarding the study protocol.

 1. How do the authors define study group A and study group B, what do they mean by healthy post-vaccination COVID-19 individuals and what do they mean by post-vaccination COVID-19 patients? What clinical manifestations will these patients have, are they related to vitamin D deficiency or adverse effects of vaccination?

 2. How will the authors demonstrate that VDR mRNA expression deficiencies and low serum vitamin D levels are not due to a vitamin D deficiency prior to vaccination?

 3. How will the study demonstrate that these VDR mRNA expression defects and vitamin D deficiency are due to the application of a specific type of SARS Cov-2 vaccine? Are there differences in the influence on vitamin D metabolism between different types of vaccines?

Is the study design appropriate for the research question?

Partly

Is the rationale for, and objectives of, the study clearly described?

Partly

Are sufficient details of the methods provided to allow replication by others?

Partly

Are the datasets clearly presented in a useable and accessible format?

Partly

Reviewer Expertise:

Infectious Diseases, Immunology, Viral Diseases

I confirm that I have read this submission and believe that I have an appropriate level of expertise to confirm that it is of an acceptable scientific standard, however I have significant reservations, as outlined above.

## References

[1] : Vitamin D and COVID-19: Clinical Evidence and Immunological Insights. *Life* .2025;15(5) : 10.3390/life15050733 10.3390/life15050733 PMC1211280640430160

# Reviewer report: Reviewer response for version 1

**DOI:** 10.5256/f1000research.157818.r285926

**Published:** 2024-06-14

**Authors:** Gaurav Kaushik

**Affiliations:** 1 School of Allied Health Sciences, Sharda University (Ringgold ID: 193167), Greater Noida, Uttar Pradesh, India

The paper proposes an investigation into the effects of COVID-19 vaccination on Vitamin D Receptor (VDR) mRNA expression and its relationship with calcium metabolism and intact parathyroid hormone (iPTH) levels. The study aims to explore the immunomodulatory effects of COVID-19 vaccines beyond their primary role in providing immunity against SARS-CoV-2. By examining the interplay among VDR mRNA expression, calcium levels, and iPTH concentrations, the research seeks to uncover potential mechanisms underlying vaccine-induced changes in immune and calcium homeostasis. This study is particularly relevant as it addresses an underexplored aspect of COVID-19 vaccination and can have significant implications for personalized vaccination strategies and the management of individuals with pre-existing calcium-related disorders.

**Major Points:**

  1. The study addresses a novel aspect of COVID-19 vaccination by focusing on VDR mRNA expression and its downstream effects on calcium metabolism and iPTH levels.

 2. The hypothesis is well-defined, aiming to explore the relationship between VDR mRNA expression and various biochemical parameters post-vaccination.

 3. The objectives are clear and align well with the overall aim of the study, providing a structured approach to investigating the proposed research questions.

**Constructive Feedback:**

 Overall, the proposed study is well-conceived and addresses a pertinent and novel question in the context of COVID-19 vaccination.

Is the study design appropriate for the research question?

Yes

Is the rationale for, and objectives of, the study clearly described?

Yes

Are sufficient details of the methods provided to allow replication by others?

Yes

Are the datasets clearly presented in a useable and accessible format?

Yes

Reviewer Expertise:

Molecular Biology, Molecular Diagnostics, Clinical Research, Molecular Genetics, Infectious Diseases.

I confirm that I have read this submission and believe that I have an appropriate level of expertise to confirm that it is of an acceptable scientific standard.
